# Emerging hemispheric asymmetry of Earth’s radiation

**DOI:** 10.1073/pnas.2511595122

**Published:** 2025-09-29

**Authors:** Norman G. Loeb, Tyler J. Thorsen, Seiji Kato, Fred G. Rose, Øivind Hodnebrog, Gunnar Myhre

**Affiliations:** ^a^Science Directorate, NASA Langley Research Center, Hampton, VA 23681-2199; ^b^Analytical Mechanics Associates, Hampton, VA 23666-6413; ^c^Center for International Climate Research, Oslo 0318, Norway

**Keywords:** Earth radiation budget, clouds, climate change

## Abstract

Twenty-four years of satellite observations from the Clouds and the Earth’s Radiant Energy System show a northern hemisphere (NH) minus southern hemisphere (SH) trend difference of 0.34 ± 0.23 Wm^−2^ dec^−1^ (5 to 95% CI) in absorbed solar radiation (ASR) and a weaker trend difference of 0.21 ± 0.21 Wm^−2^ dec^−1^ in outgoing longwave radiation. The emerging darkening of the NH relative to the SH is associated with changes in hemispheric differences in aerosol–radiation interactions, surface albedo, and water vapor changes. Cloud changes also contribute to a greater ASR hemispheric contrast, but the magnitude is small due to opposing trend differences in the tropics and extratropics. The break in hemispheric symmetry in ASR challenges the notion that clouds naturally compensate for forced hemispheric asymmetries in noncloud properties. Hemispheric (a)symmetry in radiation is linked with the atmosphere–ocean general circulation. How clouds respond to this hemispheric imbalance has important implications for future climate.

## Significance

The general circulation of the atmosphere–ocean system is closely linked with the distribution of radiant energy within the climate system. On average, the southern hemisphere and northern hemisphere (NH) reflect the same amount of solar radiation, and the NH emits more outgoing longwave radiation. Using satellite observations, we find that while both hemispheres are darkening, the NH is darkening at a faster rate. The break in hemispheric symmetry in reflected solar radiation challenges the hypothesis that hemispheric symmetry in albedo is a fundamental property of Earth. Whether the general circulation adjusts to produce a cloud distribution that restores hemispheric symmetry in albedo in the future is an open question that has important implications for future climate.

Earth’s radiation budget (ERB) is a key driver of atmospheric and oceanic circulation. On average, the southern hemisphere (SH) gains radiative energy at the top of atmosphere (TOA) while there is a net loss in the northern hemisphere (NH). This imbalance is compensated by combined atmospheric and oceanic circulations that transport energy across the equator from the SH to the NH (1–4). The hemispheric imbalance in net radiation arises because the warmer NH emits more thermal infrared radiation to space compared to the SH, while both hemispheres absorb approximately the same amount of incoming solar radiation. Since the SH and NH average incoming solar radiation is almost identical, both hemispheres must have nearly the same albedo. Hemispheric albedo symmetry has been a topic of fascination since it was first observed from satellites (5). Much speculation exists about whether this is a fundamental property of the climate system or occurs just by chance (6–8). Partitioning Earth into pairs of random halves, Voigt et al. (6) show using satellite observations of ERB from the Clouds and the Earth’s Radiant Energy System (CERES) (9) that only 3% of the random pairs exhibit hemispheric symmetry within 0.1 Wm^−2^, which is the difference between the SH and NH observed by CERES for 2000 to 2010. The distribution of clouds is a key reason for hemispheric albedo symmetry—without them the NH would be brighter than the SH (10). In response to imposed albedo changes in one hemisphere, equilibrium and transient idealized model experiments suggest that clouds compensate for hemispheric asymmetries (11–14).

Prior studies have shown that hemispheric symmetry in albedo has been persistent during the CERES period (8, 15). This has occurred in spite of a marked increase in global mean net TOA radiation (or Earth’s Energy Imbalance, EEI) resulting from a large positive trend in global mean absorbed solar radiation (ASR) that exceeds the increasing trend in outgoing longwave radiation (OLR) by more than a factor of two (16). Using 24 y of CERES data, we find emerging trends indicating that the NH is absorbing more incoming solar radiation and emitting more OLR compared to the SH. A partial radiative perturbation (PRP) analysis using additional data sources is performed to identify what properties contribute most to the hemispheric difference trends. The observational results are placed in the context of prior studies on the role of clouds and atmospheric circulation as they relate to hemispheric symmetry in ERB.

## Changes in Hemispheric TOA Fluxes.

**Fig. 1.**
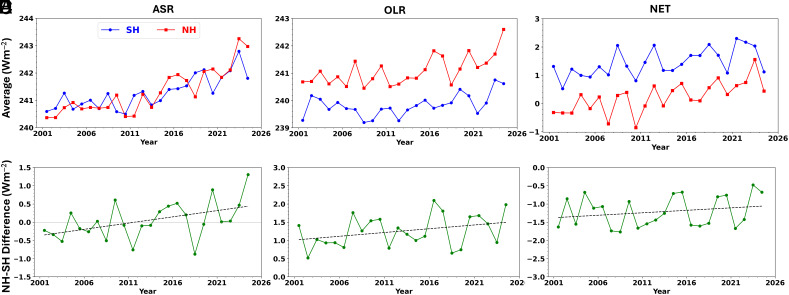
Annual means in (*A*) ASR, (*B*) OLR, and (*C*) NET for the SH and NH and NH minus SH differences in (*D*) ASR, (*E*) OLR, and (*F*) NET. Dashed lines correspond to least-squares fits to the annual mean differences.

Using TOA observations from the CERES Energy Balanced and Filled (EBAF) Ed4.2.1 product (17) for 01/2001-12/2024 (*Materials and Methods*), we find that while both the SH and NH hemispheres show increasing trends in ASR (Fig. 1*A*), the NH is darkening faster, resulting in a trend of 0.34 ± 0.23 Wm^–2^ dec^–1^ in the NH–SH ASR difference (5 to 95% CI; Fig. 1*D* and Table 1). This trend also exceeds the 2.5 to 97.5% CI, remains significant at the 5 to 95% significance level after removing endpoints, subtracting the influence of the El Niño-Southern Oscillation, and using circular block bootstrapping to determine CIs. The sign of the NH–SH ASR difference also changes—during the first 5 y of the record (2001 to 2005) the SH average ASR exceeds the NH average by 0.20 Wm^–2^ while the NH average is greater by 0.54 Wm^–2^ during the last 5 y (2020 to 2024). The large increasing trend in NH ASR is primarily due to a marked increase in the subtropics (20 to 42°N), which reaches 0.51 ± 0.25 Wm^–2^ dec^–1^ after scaling by its area fraction of the NH (a factor of 1/3, *Materials and Methods*) (*SI Appendix*, Figs. S1 and S4*A*). Both the NH and SH show an increasing trend in OLR, but radiative cooling is stronger in the NH, resulting in a NH–SH OLR difference trend of 0.21±0.21 Wm^–2^ dec^–1^, just barely within the 5 to 95% CI. This is a result of stronger radiative cooling in the NH subtropics and mid-high latitudes (*SI Appendix*, Figs. S2 and S4). As the trends in the NH–SH ASR and OLR differences correspond to radiative heating and cooling of the NH relative to the SH, respectively, they largely offset one other, resulting in a weak trend in the NH–SH NET difference (0.14 ± 0.21 Wm^–2^ dec^–1^; Table 1). This implies no significant change in combined atmosphere–ocean cross-equatorial heat transport.

**Table 1.** Trends and 5 to 95% CI in SH, NH, Globe and NH-SH annual means for 2001 to 2024. Units: Wm^−2^ dec^−1^

	SH	NH	Globe	NH-SH
ASR	0.66 ± 0.18	1.00 ± 0.32	0.83 ± 0.31	0.34 ± 0.23
OLR	0.28 ± 0.25	0.49 ± 0.21	0.39 ± 0.17	0.21 ± 0.21
NET	0.38 ± 0.21	0.52 ± 0.21	0.45 ± 0.18	0.14 ± 0.21

## Attribution of Hemispheric Trends.

**Fig. 2.**
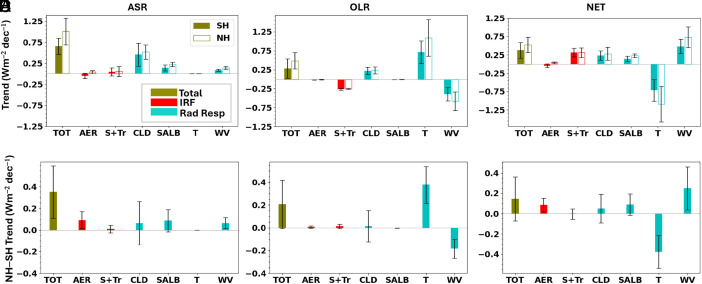
Attribution of TOA flux trends and trend differences for 2001 to 2024. Contributions from changes in AER and solar+trace gases (S + Tr) are associated with IRF, contributions from changes in clouds (CLD), surface albedo (SALB), temperature (T), and water vapor (WV) are associated with *dR*_λ_ (or equivalently, radiative response), and the sum of all contributions (*dR* or TOT) is also shown for (*A* and *D*) ASR, (*B* and *E*) OLR, and (*C* and *F*) NET. Error bars correspond to 5 to 95% CI.

**Fig. 3.**
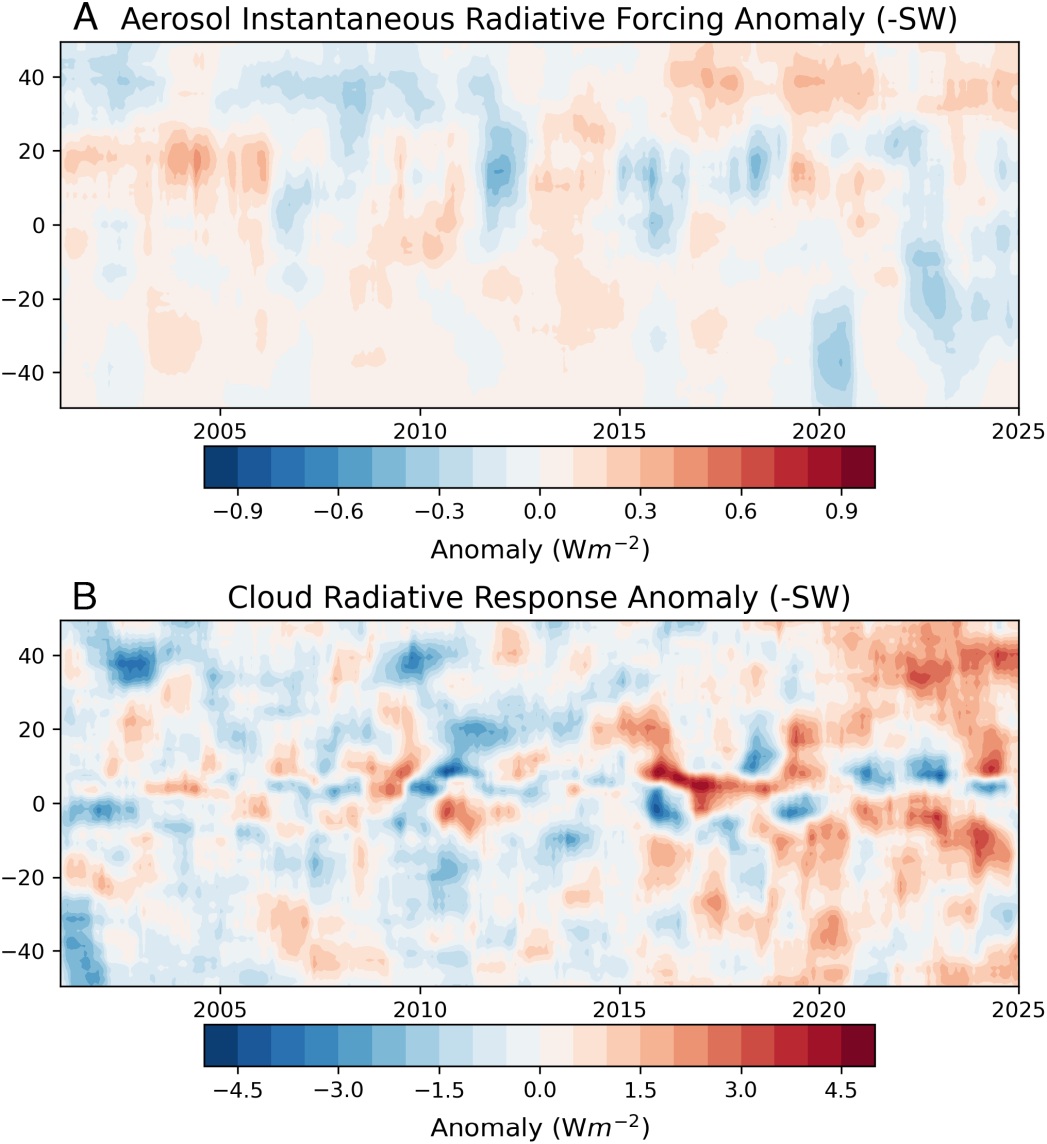
Zonal mean anomalies in (*A*) aerosol–radiation interaction IRF and (*B*)–SW cloud radiative response for 2001 to 2024.

We extend the PRP analysis used in ref. 16 (*Materials and Methods*) to examine the underlying contributions to the hemispheric trends in Fig. 1 from different atmospheric and surface variables (Fig. 2). The contributions are categorized according to whether they correspond to an instantaneous radiative forcing (IRF) or a radiative response (${\mathrm{dR}}_{\lambda }$) (*Materials and Methods*). The largest contributions to the SH and NH ASR trends are from cloud, surface albedo, and water vapor changes (Fig. 2*A*). In contrast, the largest contributions to the NH–SH ASR difference trend are from aerosol–radiation interactions, surface albedo, water vapor changes, and cloud changes (Fig. 2*D*). The relatively small cloud contribution to the NH–SH ASR difference trend is notable given its dominance for the hemispheric trends. Given that pollution has decreased since 2000 over China, the United States and Europe (18–20), and the 2019 to 2020 Australian bushfires (21, 22) and 2021 to 2022 Hunga Tonga eruption (23) in the SH happened during the latter part of the CERES record (Fig. 3*A*), the positive contribution from aerosol–radiation interactions to the NH–SH ASR difference is expected. Similarly, larger NH decreases in sea-ice concentration, snow cover, and increases in atmospheric moisture contribute to further NH darkening.

The reason for the weak hemispheric contrast in the ASR trend contribution from cloud changes is less clear. The cloud response is a result of surface-temperature mediated cloud radiative feedback and rapid cloud adjustments to greenhouse gases, aerosols, and natural forcing agents (24). It is possible that these offset one another, but we cannot tell from observations alone. To gain some insight, we use the multimodel mean climate model simulations in ref. 25 for 2001 to 2019 to examine hemispheric trends in cloud radiative effect (CRE; defined as the difference between clear and all-sky downward TOA flux) and contributions from cloud feedback, “effective radiative forcing” ERF (sum of IRF and rapid adjustments), and the associated cloud masking terms (due to variations in noncloud properties). The model simulations in *SI Appendix*, Fig. S5 show a large negative trend in the NH–SH difference in the sum of cloud feedback and cloud feedback masking ($\Delta W_{\mathrm{CRE}}$) and a positive trend in the NH–SH difference in the sum of ERF and ERF cloud-masking ($\Delta {\mathrm{ERF}}_{\mathrm{CRE}}$). As SW cloud masking trend contributions are much smaller than those from cloud feedback and ERF (figure 6 in ref. 24), these results suggest that the weak hemispheric contrast in the cloud contribution (Fig. 2*D*) arises because of compensation between cloud feedback and ERF. While a larger $\Delta {\mathrm{ERF}}_{\mathrm{CRE}}$ trend in the NH is expected due to the decrease in NH pollution since 2000, the reason for the negative trend in the NH–SH difference in $\Delta W_{\mathrm{CRE}}$ is less evident. The SH is the cloudier hemisphere, so perhaps it responds more in an absolute sense to increases in surface temperature than the NH, whose greater landmass may also buffer any hemispheric albedo decrease from reduced cloud cover.

The overall increase in longwave radiative cooling in both hemispheres is a result of contributions from temperature (surface and atmospheric temperature) and cloud changes partially offset by contributions from changes in water vapor and trace gases (Fig. 2*B*). While trace gas and cloud contributions exhibit hemispheric symmetry, stronger warming in the NH results in larger NH radiative cooling from temperature changes (Planck feedback) (Fig. 2*E*). Positive overall SH and NH trends in NET radiation occur because the combined contributions from trace gas, cloud, surface albedo, and water vapor changes overwhelm the negative Planck temperature feedback contribution (Fig. 2*C*). In contrast, NET hemispheric differences in contributions from surface albedo, temperature, and water vapor changes largely cancel one another, so that the total is close in magnitude to the contribution from aerosol-radiation changes (Fig. 2*F*).

## Hemispheric Trends in Surface Temperature, Precipitation, and CRE.

Numerous studies have suggested a strong link between hemispheric asymmetries in albedo, surface temperature, and clouds. Chiang et al. (26) showed that small high-latitude increases in ice concentration similar to those during the Last Glacial Maximum lead to a marked southward shift of the ITCZ. Kang et al. (11) used idealized climate model experiments to show and explain why asymmetric heating in the extratropics leads to latitudinal shifts in tropical rainfall toward the hemisphere that is heated more. Such shifts result from various forcing agents (12), including aerosols, and occur in coupled models as well as slab ocean models (27, 28), and apply to slab ocean simulations of global warming (29). Hwang and Frierson (30) use historical simulations from Phases 3 and 5 of the Coupled Model Intercomparison Project (CMIP3 and CMIP5) to demonstrate relationships between hemispheric asymmetries in surface air temperature, tropical precipitation, cross-equatorial atmospheric heat transport, and clouds (see their Fig. 3). They find that models with an anomalously warm NH also have greater NH tropical precipitation, stronger southward cross-equatorial atmospheric heat transport, and a stronger extratropical NH–SH difference in SW CRE (implying enhanced extratropical SH cloud reflection).

**Fig. 4.**
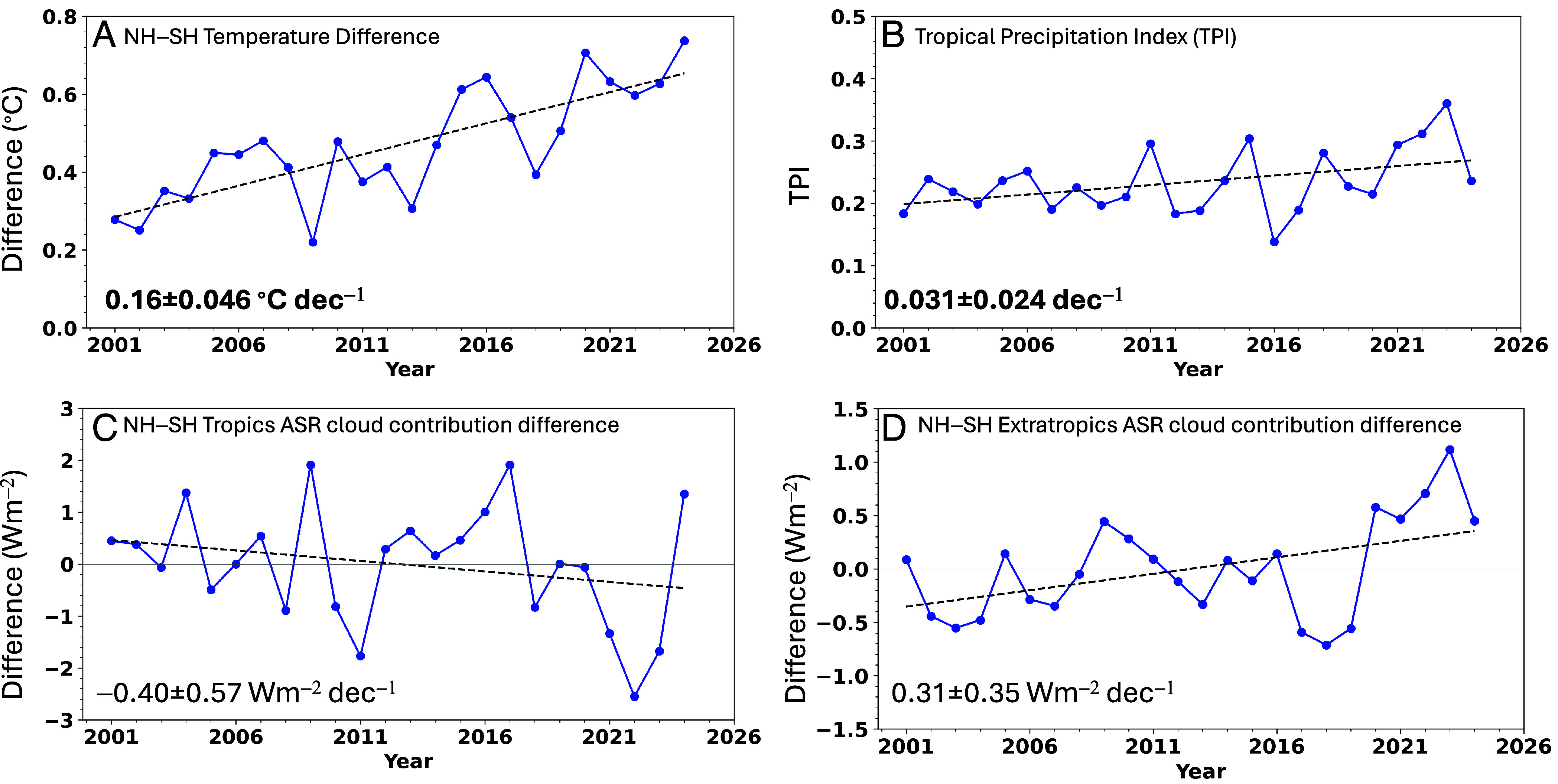
(*A*) NH–SH surface air temperature difference; (*B*) tropical precipitation index; (*C*) NH–SH tropics ASR cloud contribution difference; (*D*) NH-SH extratropics ASR cloud contribution difference for 2001 to 2024. Trends and 5 to 95% CIs indicated for each plot.

Observational trends since 2001 are qualitatively consistent with expectation based upon these modeling studies. Surface temperature in the NH is increasing faster than in the SH by 0.16 ± 0.056 °C dec^–1^ (Fig. 4*A*), and the NH tropical precipitation trend exceeds that in the SH, as indicated in Fig. 4*B* by a positive trend in tropical precipitation index, defined as the equator to 20°N minus equator to 20°S tropical mean precipitation normalized by the mean for 20°S-20°N. The increasing trend in the NH–SH tropical precipitation difference is qualitatively consistent with a decreasing trend in the cloud contribution to the NH–SH tropical ASR difference (Fig. 4*C*)—we find that SH tropical precipitation decreases relative to the NH tropics and SH tropical ASR resulting from cloud changes increases relative to the NH tropics, implying progressively less precipitation and clouds in the SH tropics relative to the NH tropics with time. In contrast, the cloud contribution to the NH–SH difference in ASR for the extratropics (20°-90°) shows the opposite trend, with the NH clouds reflecting less compared to those in the SH (Fig. 4*D*). It is noteworthy that the tropical and extratropical NH–SH difference anomalies are most pronounced after 2020 as clearly seen in the Hovemoller plot in Fig. 3*B*. The opposite hemispheric cloud contribution trends in the tropics and extratropics largely cancel on another, leading to near symmetry in the cloud ASR trend contribution over the entire NH and SH.

## Discussion and Conclusions

While the increasing difference between NH and SH ASR in the CERES record does not rule out the possibility that mechanisms exist to minimize hemispheric differences in albedo in some fundamental way (6, 7, 31), it does raise questions about how quantitatively robust this possible constraint might be. The 5-y average NH–SH ASR difference has grown from –0.20 Wm^–2^ to 0.54 Wm^–2^ in 19 y. While climate models can in principal estimate how much larger this difference can get, prior studies show discrepancies of ±5 Wm^–2^ in NH–SH SW TOA flux difference among models (32, 33), casting doubt on their reliability for this purpose. Since the NH darkening (relative to the SH) due to noncloud property changes (aerosol–radiation interactions, surface albedo, water vapor) is not compensated by cloud changes, this suggests that there may be a limit to clouds’ role in maintaining hemispheric symmetry in albedo. If clear-sky hemispheric albedo asymmetry is a transient feature of Earth’s climate (31), our results suggest the same may be true for all-sky, at least to some extent, which we currently cannot quantify. The hemispheric difference in surface warming and surface albedo in response to increasing CO_2_ forcing seen in climate model simulations (33) together with any further hemispheric changes in aerosol suggests we should see an increase in hemispheric albedo asymmetry in the future. However, if clouds compensate for hemispheric asymmetry (e.g., through circulation changes), but do so over a longer timescale, the trend in the NH–SH ASR difference may reach some upper limit.

In addition to NH darkening and warming relative to the SH, the NH tropics are getting wetter, which suggest changes in large-scale atmospheric circulation are occurring. Tselioudis et al. (34) show evidence for a poleward displacement of the midlatitude storm zone and narrowing of the ITCZ, but they do not examine hemispheric differences. Hadas et al. (35) argue that in a warmer climate reduced baroclinic activity due to Arctic Amplification should lead to weaker cloud albedo in the NH midlatitudes, but how cloud albedos in the SH midlatitudes will change is more uncertain. Our observational results suggest the NH extratropics will likely darken relative to the SH extratropics (Fig. 4*D*), but the short observational record precludes a definitive conclusion. Clearly, a longer observational record is needed to precisely monitor the evolution of TOA radiation, clouds, and atmosphere–ocean circulation.

## Materials and Methods

### Satellite Data.

We consider CERES EBAF Ed4.2.1 for 01/2001-12/2024. EBAF provides monthly mean TOA and surface SW, LW, and net radiative fluxes on a 1° × 1° grid along with solar irradiance measurements and imager-derived cloud properties (17, 36). An objective constrainment algorithm (37) is used to adjust SW and LW TOA fluxes within their ranges of uncertainty to anchor global net TOA flux to an in situ estimate of the global mean EEI from mid-2005 to mid-2015 (38). Use of this approach to anchor the satellite-derived EEI and all other processing steps used to produce EBAF have no impact on the variability or trends between the hemispheres. ASR is determined from the difference between spatially and temporally averaged monthly solar irradiances and reflected SW fluxes. Annual means are determined for each calendar year from weighted averages of monthly data where the weights are given by the number of days in a month, accounting for leap years. To ensure hemispheric symmetry in solar irradiance for each year, each December average is weighted by 31.25 d for nonleap years and 30.25 d for leap years. Annual anomalies are calculated from the difference between individual years and the average of all years. Trends are determined from a least-squares regression fit to annual anomalies with uncertainties given as 5 to 95% CI using the approach described in ref. 39. Measurement uncertainty is estimated by comparing annual mean NH–SH TOA flux trends from SSF1deg-Aqua, SSF1deg-Terra, SYN1deg-Terra+Aqua+GEO, and EBAF Ed4.2.1 for the common Terra and Aqua period of 2003 to 2021, when these satellites maintained a fixed mean local time (*SI Appendix*, Table S1). The EBAF NH–SH TOA flux trends are consistent with those from SSF1deg-AQU, SSF1deg-TER, SYN1deg to ~0.03 Wm^−2^ dec^−1^ for SW and 0.035 Wm^−2^ dec^−1^ for LW. This is approximately an order-of-magnitude smaller than the NH–SH trend uncertainty. Hemispheric average TOA fluxes are determined by applying geodetic zone weights to 1° zonal averages. Each hemisphere is further subdivided into zones corresponding to the tropics (0°–20°), subtropics (20°–42°), and mid-high latitudes (42°–90°). These latitude ranges are selected because they cover approximately equal area. Averages for these zones are divided by 3 to show their contribution to the hemispheric mean (*SI Appendix*, Figs. S1–S4).

### PRP Analysis.

The PRP method (40) is a way of quantifying anomalies in TOA radiation associated with individual variables. Here, we use the PRP method as described in ref. 41 and implemented in ref. 16. For noncloud contributions, the effect on the flux (δF) due to some perturbation Δx of variable x is computed through radiative transfer model calculations initialized using gridded monthly mean variables with variable x perturbed by Δx, given by the deseasonalized monthly mean anomaly of x. We use a centered finite difference obtained by averaging the backward and forward finite differences. To determine cloud contributions, the anomaly in CRE, the difference between all-sky and clear-sky TOA flux, is adjusted for cloud masking of changes in noncloud properties (24).

The radiative transfer model used is the NASA Langley Fu–Liou radiative transfer model (42). Input variables include skin temperature, profiles of temperature and water vapor, surface albedo, aerosols, trace gases (ozone, carbon dioxide, methane, nitrous oxide, CFC-11, CFC-12, and HCFC-22), and incoming solar irradiance. The input variables are those used in the surface flux calculations in CERES EBAF Ed4.2.1 (36) consisting of adjusted input values that are “tuned” to force a match between computed and observed EBAF monthly mean all-sky and clear-sky TOA fluxes through an objective constrainment algorithm (42, 43). As a result, the sum of all PRP contributions closely track EBAF TOA. Comparing hemispheric trends for 2001 to 2024 from the PRP method and EBAF yields differences <0.03 Wm^–2^ dec^–1^. Furthermore, based upon direct comparisons between trends determined from CERES Terra and CERES Aqua monthly CRE anomalies for 09/2002-03/2020, Loeb et al. (16) estimate the trend uncertainty in CRE due to instrument drift to be <0.085 W m^−2^ dec^−1^, which is a factor of five smaller than the trend uncertainty associated with CRE internal variability.

The skin temperature, surface pressure, and profiles of temperature, water vapor, and ozone are from the MERRA-2 (44). Carbon dioxide concentrations are obtained from the AIRS, version 5 level 3, carbon dioxide product (45). Other trace gases are obtained from National Oceanic and Atmospheric Administration Earth System Research Laboratory Global Monitoring Division (46). Aerosol optical depths (at 0.55 µm) and vertical distributions of seven aerosol types are from the Model of Atmospheric Transport and Chemistry Moderate Resolution Imaging Spectroradiometer aerosol assimilation product (47). Spectral surface albedos are determined using surface-type-based lookup tables (48) for the spectral shapes that are scaled by the broadband surface albedo determined from the CERES surface albedo history map (48).

### Surface Temperature and Precipitation Data.

To examine hemispheric trends in surface temperature, we use the Met Office Hadley Centre/Climatic Research Unit global surface temperature dataset (HadCRUT5 version 5.0.2.0) (49). For precipitation, we use the Global Precipitation Climatology Monthly product (50).

### Forcing-Feedback Framework.

The different PRP atmosphere and surface contributions are categorized according to the widely used forcing-feedback framework (51), as applied to observations (52):

$$dR=\mathrm{IRF}+{\mathrm{dR}}_{\lambda },$$

where *dR* is the change in net TOA radiation, IRF, and ${\mathrm{dR}}_{\lambda }$ is the change in net TOA radiation associated with surface-temperature mediated radiative feedbacks and rapid adjustments to radiative forcing that are independent of surface temperature. IRF corresponds to perturbations in TOA radiation of anthropogenic (e.g., atmospheric composition, land use change) and natural (e.g., volcanic activities and solar variations) origin. IRF differs from ERF commonly used in climate model analyses by the rapid adjustments term, which in observational analyses is included as part of ${\mathrm{dR}}_{\lambda }$. In climate model simulations, rapid adjustments are easily separated from surface-temperature-mediated radiative feedbacks and thus are bookept as part of the ERF forcing term (24).

IRF includes contributions from solar variations, trace gases, and aerosol–radiation interactions. Surface temperature-mediated radiative feedbacks are from surface albedo, clouds, temperature, and water vapor. Importantly, aerosol–cloud interactions, normally considered a forcing contribution in model analyses, are not easily separated from cloud radiative feedbacks in observations and thus are included in the radiative response term (see also ref. 52).

## Supplementary Material

Appendix 01 (PDF)

## Data Availability

All study data are included in the article and/or *SI Appendix*.
