# Supplementary material for: Emerging hemispheric asymmetry of Earth’s radiation
Source: Proc Natl Acad Sci U S A. 2025 Sep 29;122(40):e2511595122. doi: 10.1073/pnas.2511595122 (PMC12519167; doi:10.1073/pnas.2511595122)
Supplement: Supplementary file 1 — Appendix 01 (PDF) [file pnas.2511595122.sapp.pdf]

PNAS

Supporting Information for

Emerging Hemispheric Asymmetry of Earth's Radiation

Norman G. Loeb[1*], Tyler J. Thorsen[1], Seiji Kato[1], Fred G. Rose[2], Øivind Hodnebrog[3], and Gunnar Myhre[3]

[1]NASA Langley Research Center, Hampton, VA USA.

[2]Analytical Mechanics Associates, Hampton, VA, USA.

[3]Center for International Climate Research (CICERO), Oslo, Norway.

*Norman G. Loeb, NASA Langley Research Center, Hampton, VA 21 Langley Boulevard, Hampton, VA 23681; (757) 870-1684.

Email: norman.g.loeb@nasa.gov

This PDF file includes:

Figures S1 to S5
Table S1

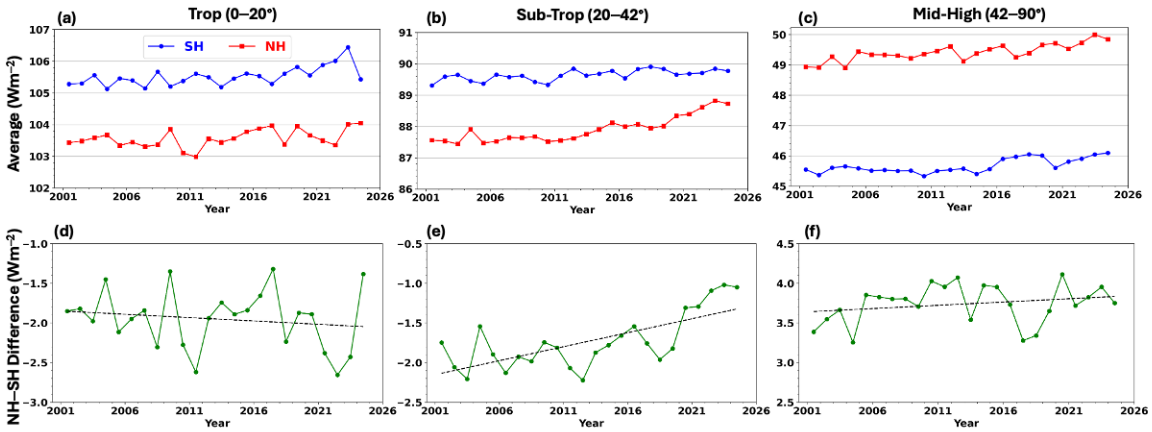

Figure S1 Area fraction weighted annual mean SH and NH ASR (top row) and NH-SH ASR difference (bottom row) for the (a, d) Tropics (0-20°), (b, e) Sub-Tropics (20-42°) and (c, f) Mid-High latitudes (42-90°). Dashed lines correspond to least-squares fits to the annual mean differences.

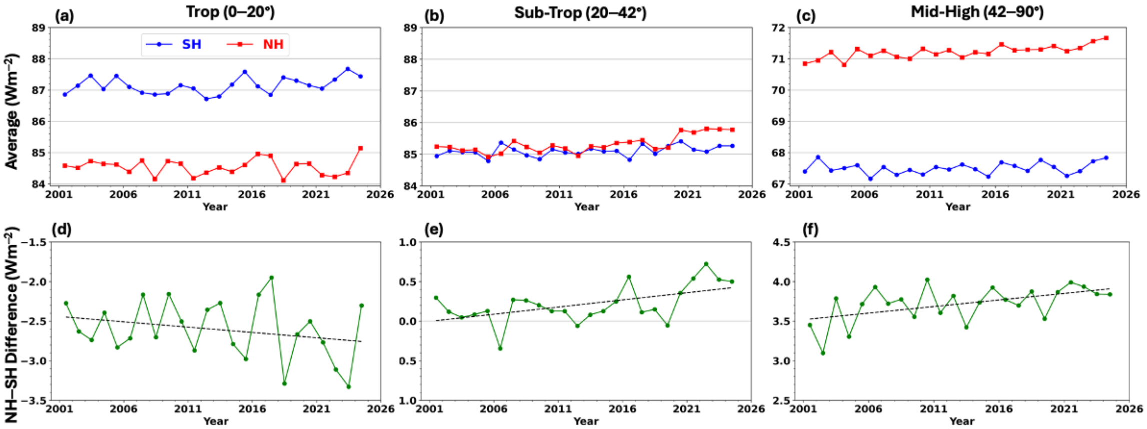

Figure S2 Same as Figure S1 but for OLR.

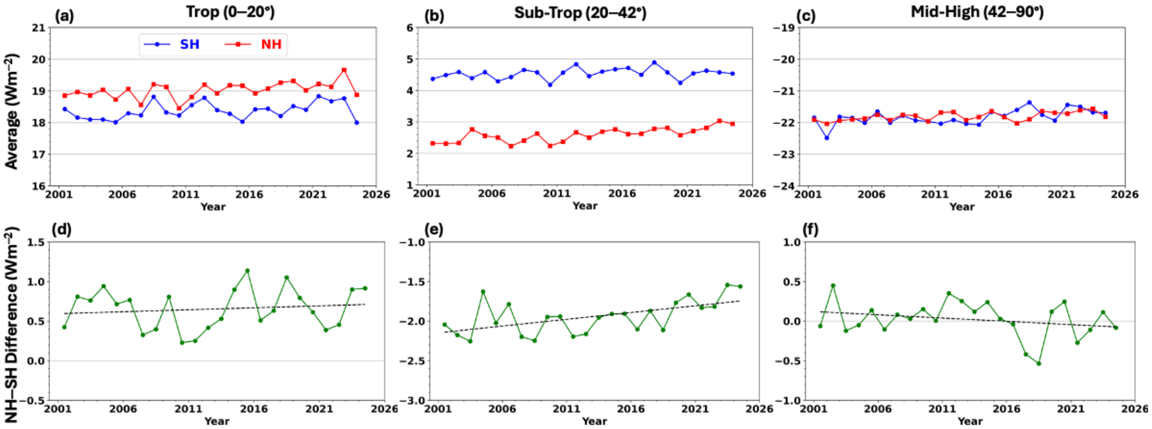

Figure S3 Same as Figure S1 but for NET.

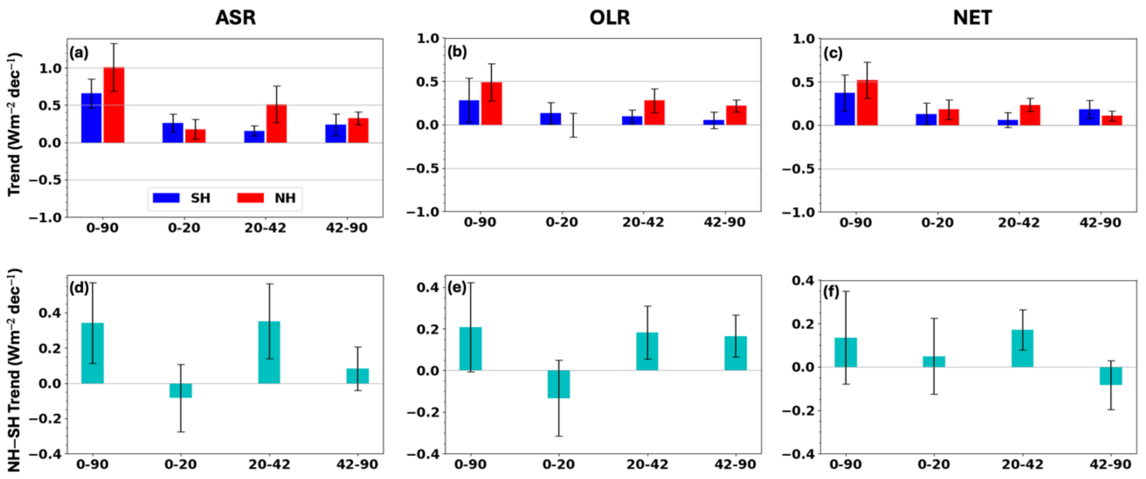

Figure S4 SH and NH trends and area fraction weighted contributions from the tropics (0-20°), sub-tropics (20-42°) and mid-high latitudes (42-90°) for (a) ASR, (b) OLR and (c) NET. (d-f) NH–SH difference trends corresponding to (a-c). Error bars correspond to 5%-95% confidence intervals.

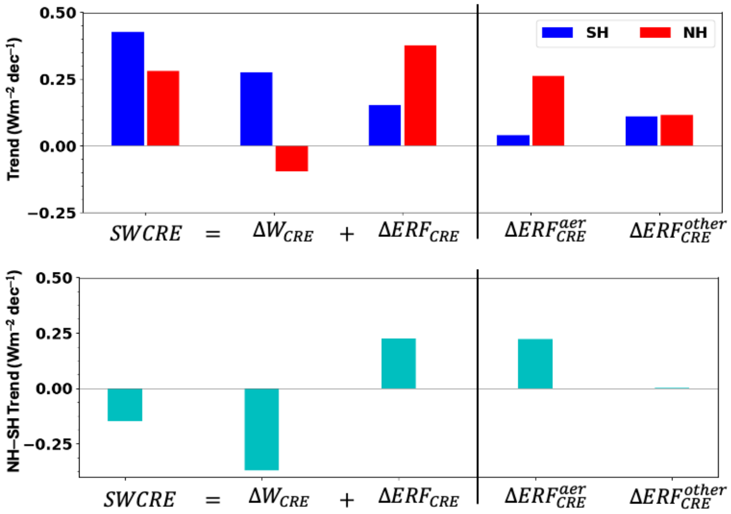

Figure S5 Global climate model simulations of (a) SH and NH trends in SW CRE and contributions from sum of cloud feedback and cloud masking terms (ΔW_{CRE}) and effective radiative forcing (ERF) and ERF cloud-masking terms (ΔERF_{CRE}). Also shown are trends for anthropogenic aerosol (ΔERF_{CRE}^{aer}) and other forcing (ΔERF_{CRE}^{other}) components of ΔERF_{CRE}. (b) Same as (a) but for NH-SH. Variable definitions closely follow those in Raghuraman et al. (2023). As described in Hodnebrog et al. (2024), climate model simulations correspond to multi-model mean values for 2001-2019 and use observed sea-surface temperatures and sea-ice concentration.

Table S1 NH−SH trends in SW and LW TOA flux for 2003-2021. Also provided are the uncertainties in EBAF NH−SH trend differences (at 90% significance level) determined by comparison with the other CERES products.

CERES Product	SW NH−SH Trend (Wm^{-2} dec^{-1})	LW NH−SH Trend (Wm^{-2} dec^{-1})
SSF1deg-AQU	-0.2104	0.1340
SSF1deg-TER	-0.2324	0.1660
SYN1deg	-0.2447	0.1749
EBAF	-0.2274	0.1905
**EBAF Error**	**0.030**	**0.035**
